# The Effect of High Levels Carbohydrate on Intestinal Microbiota, Metabolites, and Health of Common Carp (*Cyprinus carpio* L.)

**DOI:** 10.1155/2024/7631021

**Published:** 2024-10-23

**Authors:** Jinrui Xiong, Liping Yang, Luming Wang, Shaoyang Zhi, Mengjuan Zhao, Chunchu Xu, Leya Qu, Xiaorui Guo, Xiao Yan, Chaobin Qin, Guoxing Nie

**Affiliations:** College of Fisheries, Henan Normal University, No 46 Jianshe Road, Xinxiang 453007, China

**Keywords:** *Cyprinus carpio* L., dietary carbohydrate, intestinal health, intestinal microbiota, microbial metabolites

## Abstract

Long-term consumption of high-carbohydrate feed may adversely affect intestinal health of fish; however, the underlying roles remain ambiguous. This study examined the effects of varying carbohydrate levels on the intestinal flora of common carp and assessed how microbial metabolites influence intestinal health. Two hundred seventy common carps were chosen and distributed randomly into three groups that fed diets containing starch at levels of 15% (low-carbohydrate diet [LCD]), 28% (medium-carbohydrate diet [MCD]), and 45% (high-carbohydrate diet [HCD]) for 60 days. A significant increase in final body weight, weight gain rate, and specific growth rate within the MCD group, while feed conversion ratio exhibited a decrease in comparison to the other groups (*p* < 0.05). Feeding with a HCD led to decreased activity of catalase and increased malondialdehyde content, which was consistent with reverse transcription-quantitative real-time polymerase chain reaction (RT-qPCR) analysis results (*p* < 0.05). Specifically, the RT-qPCR results revealed that HCD treatment significantly upregulated *il1β*, *il6*, and *il8* transcript levels. Whereas, the *il10* messenger RNA (mRNA) was markedly reduced in comparison to the LCD group. Furthermore, the HCD group exhibited an increased abundance of *Proteobacteria*, accompanied by a reduction in *Fusobacteria* abundance, and also revealed an upsurge in opportunistic pathogenic bacteria, such as *Aeromonas* and *Shewanella*. The correlation analysis demonstrated negative correlations of anti-inflammatory active substances such as fucoxanthin, (S)-reticuline, hecogenin, and uridine with *Aeromonas*, but positive correlations with *Luteolibacter*. In summary, dietary carbohydrates might mediate intestinal flora to regulate their metabolites and affect intestinal inflammatory response.

## 1. Introduction

Carbohydrates are widely used as important macronutrients to provide energy and carbon skeleton for animals due to their cost-effectiveness and the protein-sparing effects in the feed [1, 2]. However, excessive carbohydrate intake may lead to poor growth performance, liver fat accumulation, and decreased disease resistance [3]. The utilization of carbohydrates by fish is intricately linked to their feeding patterns; carnivorous fish exhibit the least proficiency in utilizing carbohydrates, while omnivorous and herbivorous fish exhibit better abilities in this regard. Common carp (*Cyprinus carpio* L.) is a widely cultured omnivorous fish in China. The production of common carp in China reached 2.843 million tons in 2022 [4]. The carbohydrate requirement in juvenile common carp is studied by Guo [5], and it is found that 25%–40% carbohydrate is suitable. Furuichi and Yone [6] indicated that the growth performance of common carp was decreased when carbohydrates reached 40%. Other studies have shown that a high intake of corn starch in the diet can have negative effects on various aspects of health [7–9]. These effects include a decrease in food intake, an increase in the accumulation of lipids in the hepatopancreas and the whole body, and the potential development of metabolic disorders, decreased disease resistance and poor growth performance, which was also reported in other omnivorous fish, such as Tambaqui (*Colossoma macropomum*), Nile tilapia (*Oreochromis niloticus*), and Gibel carp (*Carassius auratus gibelio*) [7–9].

The intestinal tract plays a crucial role in nutrient uptake as it is the largest interface connecting the external environment to the animal body. Gut microbiota refers to the microbial community residing in the intestines of the host and plays a vital role in mucosal immunity and in physiological dysfunction by high starch ingestion [10]. Over the past few decades, there has been a growing focus on the impact of nutrition on the gut microbiota and its implications for the health of both humans and aquatic animals [11, 12]. The meaningful question now is to clarify the interaction between different types of nutrition and gut microbiota composition [13]. Long-term consumption of low-fiber foods may decrease the abundance of Firmicutes, Bacteroidetes, and butyric acid, negatively impacting intestinal health [14]. Moreover, it was found that the abundance of *Proteobacteria* increased in the gut microbiota of the high-starch group in largemouth bass (*Micropterus salmoides*) [15]. Similarly, research on golden pompano (*Trachinotus ovatus*) revealed an increase in pathogenic bacteria *Photobacterium* and *Mycoplasma* in the high starch group [16]. Furthermore, a study on tilapia found that *Firmicutes* was significantly higher in the high starch group [17]. These findings highlight the impact of dietary starch levels on the composition of intestinal flora in fish. However, recent studies have mainly focused on examining the effects of carbohydrates on the composition change of the microbial communities at the phylum level.

The constituents of the diet not only influence the composition of the microbiota but also impact its metabolites. During the fermentation process of undigested substances from external sources and compounds provided by the host's body, metabolites such as short-chain fatty acids, branched-chain amino acids, and bile acids are formed. This demonstrates the intricate relationship among diet components, microbiota composition, and metabolite production [18]. Acting as signal molecules, these metabolites play a crucial role in connecting gut microbiota with the host's physiological functions [19]. With technological advancements, it has become convenient to collect gut DNA sequences and metabolites. However, there is currently no reported research on how the association between gut metabolites and gut microbiota affects immunity and antioxidant activity. In Chinese perch (*Siniperca chuatsi*), a high-carbohydrate diet (HCD) was found to decrease gut microbiota diversity while increasing intestinal harmful bacteria and reducing gut immunity [20]. To date, limited research conducted on the combined impact of a HCD on the intestinal microbiota community, the metabolites produced by microbiota, and the immune system in the intestine.

In this study, three diets with different levels of carbohydrates (15%, 28%, and 45%) were used in a 60-day culture experiment to understand the impact of carbohydrate levels on growth performance, and the composition of intestinal bacteria, metabolites, and immune responses in common carp. The results will be useful to identify specific probiotics or prebiotics that can enhance the fish's tolerance toward HCDs by understanding how intestinal microbiota and metabolites influence gut immunity and health.

## 2. Materials and Methods

### 2.1. Ethical Statement

All fish experiments adhered to the protocols and procedures of the Laboratory Animals of Henan Normal University.

### 2.2. Experimental Diets and Feeding Trial

Table 1 displays the experimental diets. The basal diet consists of 35.31% crude protein and 6.63% crude lipid. To conduct the feeding trial, three carbohydrate-supplemented diets were formulated with varying levels of starch: 15% (low-carbohydrate diet [LCD]), 28% (medium-carbohydrate diet [MCD]), and 45% (HCD). Feed raw materials were purchased from Luohe Zhengda Feed Co., Ltd. Before combining with soybean oil and distilled water (H_2_O) (equivalent to approximately 30% of the dry weight), the dry raw ingredients underwent pulverization and sieving through a screen with an aperture size of 0.150 mm. Next, the ingredients were thoroughly mixed and introduced into a pellet machine (KL-125B) for cold extrusion, utilizing a die with a diameter of 2 mm. Following the extrusion process, the diets were dried in a controlled airflow environment at a temperature of 25°C until they reached a moisture content of approximately 10%, after which they were stored at −20°C until use.

The common carp used in the experiment were purchased from the Henan Academy of Fisheries Sciences and belonged to the same family. The experiment was conducted at the Aquatic Animal Nutrition R&D and Pilot Base of Henan Normal University (Luohe, Henan, China). A total of 270 juvenile common carp (initial weight 8.82 ± 0.01 g) were randomly distributed in nine cages, and triplicate cages were randomly assigned to each treatment. Prior to the initiation of the feeding trial, the juvenile carp belonging to the same lineage were acclimatized under controlled conditions for a duration of 2 weeks. During this acclimation period, they were provided with the MCD group's diet. The carp were then subjected to thrice-daily feedings (8:00, 12:30, and 17:30) with a ration equivalent to 3% of body weight, for a total of 60 days. Once every week, the feed intake was adjusted based on the carp's body weight. Throughout the experiment, various key H_2_O parameters were monitored. The H_2_O temperature ranged between approximately 28–33°C, a dissolved oxygen level of 6.0 to 7.0 mg/L was maintained, the pH value was maintained within the range of 7.5 to 8.0, ammonia nitrogen content was less than 0.2 mg/L, and nitrite content is less than 0.1 mg/L. The experiment was conducted under natural photoperiodic conditions.

### 2.3. Sample Collection and Analysis

After the breeding trials, the carps were fasted for 24 h before collection. Subsequently, they were subjected to anesthesia using 55 mg/L MS-222 (Aladdin, China) and had their weight and length measured. Blood samples were promptly taken from the tail vessels of nine fish per cage, utilizing 2.5 mL germ-free syringes (Klmediacal, China), slowly poured into a 2.0 mL sterile enzyme-free EP tube, and stored at 4°C overnight. Serum was obtained following centrifugation of the processed samples (3500 g, 10 min, and 4°C). The hepatosomatic index (HSI) and visceral somatic index (VSI) were measured after the dissection of the fish.

After blood collection, intestinal tissue samples were promptly obtained and snap frozen in liquid nitrogen for storage at −80°C, facilitating biochemical analysis and total RNA extraction. The intestinal tissues were fixed in 4% paraformaldehyde. The intestine contents were collected by scraping method and frozen in liquid nitrogen for gut microbiota and metabolites analysis.

### 2.4. Calculation of Growth Performance of Common Carp

The growth performance, feed utilization, and visceral parameters were evaluated using a standardized calculation formula. Additionally, the survival rate (SR) was determined. The following indicators were evaluated: initial body weight (IBW), final body weight (FBW), weight gain rate (WGR), specific growth rate (SGR), condition factor (CF), HSI, VSI, and SR. The calculation formula is as follows: WGR = (FBW − IBW)/IBW × 100, SGR = (Ln FBW − ln IBW)/breeding days, CF = FBW/body length^3^, HSI = wet weight of hepatopancreas/FBW × 100, VSI = wet weight of viscera/FBW × 100, and SR = number of surviving experimental fish/total number of experimental fish × 100.

### 2.5. Serum and Tissue Biochemical Parameter Measurements

The levels of serum glucose (GLU), triglyceride (TG), lactic acid (lactate), total cholesterol (TC), high-density lipoprotein-cholesterol (HDL-C), and low-density lipoprotein-cholesterol (LDL-C) were determined. In addition, intestinal catalase (CAT), superoxide dismutase (SOD) activities, total antioxidant capacity (T-AOC), and the content of malondialdehyde (MDA) were assessed using commercially available kits obtained from Nanjing Jiancheng Bioengineering Institute. The measurement steps followed the instructions of the respective kit protocols, and data acquisition was performed using the AU-5800 automatic biochemical analyzer manufactured by Beckman, United States.

### 2.6. RNA Extraction and Reverse Transcription-Quantitative Real-Time Polymerase Chain Reaction (RT-qPCR)

The gut tissue sample, weighing approximately 80 mg, underwent total RNA isolation with the RNAiso Plus reagent (Takara, Japan). The concentration of RNA was then assessed using NanoDrop 2000 (Thermo, United States), and all readings at 260/280 nm fell within the range of 1.9 and 2.1. The integrity of the RNA was further tested using 1% agarose gel electrophoresis. The cDNA synthesis was performed using the PrimeScript RT Kit (Takara, Japan). Primers, outlined in Table 2, were designed by Primer 6.0 software. The qPCR amplification was carried out with SYBR Green I (Vazyme, China) on a LightCycler 480 II instrument (Roche, Germany). The qPCR program commenced with the denaturation step at 95°C for 3 min, followed by 40 cycles of denaturation at 95°C for 12 s, annealing at 57°C for 12 s, and extension at 72°C for a duration of 25 s. The analysis of relative fold changes in genes was conducted utilizing the comparative 2^−*ΔΔ*Ct^ method, with normalization to the reference gene known as 18S rRNA [21].

### 2.7. Histology Analysis

The histological intestinal tissues were all fixed in a 4% paraformaldehyde solution, dehydrated using a series of ethanol solutions, and cleared with xylene. Following this, the samples were embedded in paraffin wax and subsequently sectioned to a thickness of 5 µm. Hematoxylin and eosin (H&E) staining was performed, allowing for examination under a light microscope (Zeiss, Germany). Eventually, the desired views were captured through photomicrographs.

### 2.8. Microbiota Sequencing and Analysis

The total genomic DNA of the intestinal contents was extracted by the OMEGA Soil DNA Kit (M5635-02) (Omega Bio-Tek, Norcross, GA, United States). The V3-V4 variable region of the 16S rRNA gene of intestinal bacteria was amplified by PCR. After purification and quantification, the products were sequenced on the Illumina NovaSeq 6000 (Shanghai Personal Biotechnology Co., Ltd., China). Then, the QIIME2 software was employed to obtain amplicon sequence variants (ASVs) clustered with 97% similarity. The alpha diversity including Chao1, Shannon, and Simpson indices and beta diversity were calculated through the R language ggplot2 package and QIIME2 software. Additionally, the weighted UniFrac distance was used in the principal coordinates analysis (PCoA).

### 2.9. Intestinal Metabolites Analysis

The untargeted metabolomics analysis was utilized to determine the metabolic differences in intestine of common carp after fed with different carbohydrate levels diet. Metabolites were extracted by the mix of methanol, acetonitrile, and H_2_O [22]. Six samples of each group were extracted for further analysis. The separation of the supernatants was analyzed using a Vanquish UHPLC (ultra-high performance liquid chromatography) system (Thermo Fisher Scientific). The original data were converted into mzXML (extensible markup language) format by ProteoWizard software. Utilize R packages to perform principal component analysis (PCA), partial least squares discriminant analysis (PLS-DA), and orthogonal projection to latent structures-discriminant analysis (OPLS-DA) on both the HCD and LCD groups. The differential metabolites were identified by the variable importance in the projection (VIP) and *p*-value (VIP > 1 and *p*-value < 0.05).

### 2.10. Data Analysis

The software SPSS Statistics 26.0 was utilized to perform a one-way analysis of variance (ANOVA), while Duncan's method was applied for conducting multiple comparisons. Mean values ± standard error of the mean (SEM) was presented to depict the results, with significance being determined at *p* < 0.05. Spearman's correlation coefficient tests were used for the correlation among the differential metabolites, intestinal microflora at the genus level, and immune-related genes. Moreover, data visualization was accomplished through the utilization of GraphPad Prism 9.

## 3. Results

### 3.1. Growth Performance of Common Carp Fed With Different Carbohydrate Levels

After the feeding trial, the growth performance in the MCD group exhibited superior compared to the other groups. The FBW, WGR, and SGR were significantly higher (*p* < 0.05) while the feed conversion ratio (FCR) was the lowest (*p* < 0.05; Table 3) in MCD group. Furthermore, the CF, HSI, and VSI exhibited a significant increase in the HCD group (*p* < 0.05), with no impact on the SR (*p* > 0.05; Table 3).

### 3.2. Serum Biochemical Parameters

The impact of carbohydrates on the serum biochemical parameters of common carp is presented in Table 4. The results indicate that the levels of serum GLU, TG, and HDL-C were significantly higher in the HCD group compared to the LCD group (*p* < 0.05; Table 4). In addition, the MCD group exhibited significantly higher levels of serum TG, HDL-C, and TC compared to the LCD group (*p* < 0.05; Table 4). However, no differences were observed in lactate and LDL-C (*p* > 0.05; Table 4).

### 3.3. The Intestine Antioxidant Status

**Figure 1:**
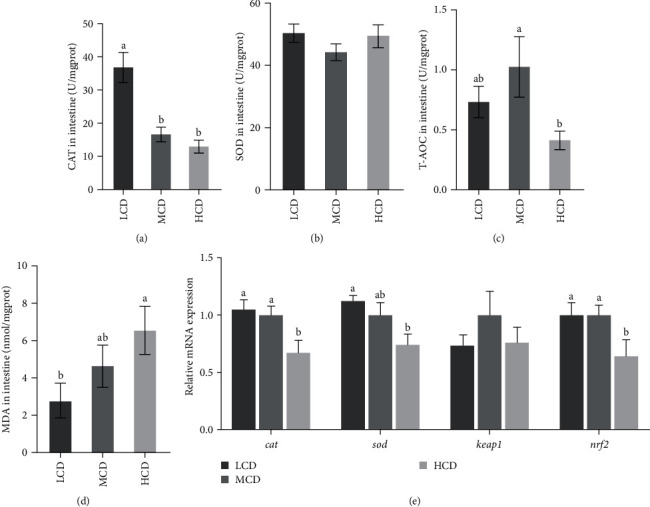
Effects of carbohydrate on antioxidant parameters in the intestine of common carp fed with different carbohydrate levels in diets (*n* = 12). (A) CAT; (B) SOD; (C) T-AOC; (D) MDA; and (E) the mRNA relative expression levels of antioxidant-related genes were measured and presented as means ± SEM (*n* = 8). Different letters indicate significant differences among all the groups (*p* < 0.05). CAT, catalase; HCD, high-carbohydrate diet; Keap1, the Kelch-like ECH-associated protein 1; LCD, low-carbohydrate diet; MCD, medium-carbohydrate diet; MDA, malondialdehyde; mRNA, messenger RNA; nrf2, nuclear factor erythroid-2- related factor 2; SOD, superoxide dismutase; T-AOC, total antioxidant capacity.

The activity of intestinal antioxidant enzymes was found to be influenced by the level of dietary carbohydrates. The T-AOC and CAT activity exhibited a significant reduction in the HCD group (*p* < 0.05; Figure 1), while the MDA content showed a significant increase (*p* < 0.05). Furthermore, the transcript level of antioxidant genes, such as *sod*, *cat*, and *nrf2*, was significantly decreased in response to a HCD (*p* < 0.05; Figure 1).

### 3.4. Expression of the Genes Related to Intestinal Immune Barrier

**Figure 2:**
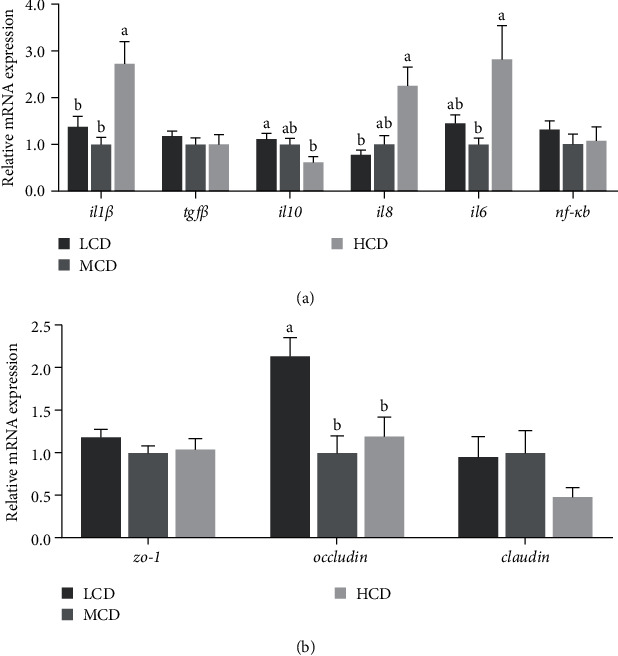
Effects of dietary carbohydrate levels on intestinal immune barrier gene expression levels in common carp (*n* = 8). (A) Inflammation-related genes; (B) tight junction-related genes. Each value is expressed as the means ± SEM, and various letters represent significant differences among all the groups (*p* < 0.05). HCD, high-carbohydrate diet; LCD, low-carbohydrate diet; MCD, medium-carbohydrate diet.

The effects of different carbohydrate levels on the expression of immune barrier-related in intestine are presented in Figure 2. The genes expression of pro-inflammatory factors (*il1β*, *il6*, and *il8*) was significant enhanced in the HCD group (*p* < 0.05). Conversely, the anti-inflammatory factor *il-10* was notably reduced in HCD group (*p* < 0.05). Furthermore, there was a notable decrease in the messenger RNA (mRNA) levels of the tight junction protein *occludin* in the HCD group (*p* < 0.05).

### 3.5. Intestine Histomorphology

**Figure 3:**
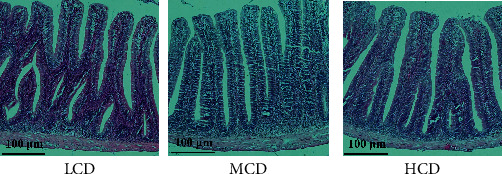
Effects of dietary carbohydrate levels on intestine under a light microscope (H&E stain). Magnification: 100x. H&E, hematoxylin and eosin; HCD, high-carbohydrate diet; LCD, low-carbohydrate diet; MCD, medium-carbohydrate diet.

The histology of the intestine and the corresponding statistical parameters is shown in Figure 3 and Table 5, respectively. The height of villus and muscle layer thickness of the intestine is markedly higher in the HCD and MCD groups compared to the LCD group (*p* < 0.05). Additionally, the intestinal villus width exhibited a significant increase in the MCD group compared to both the HCD and LCD groups (*p* < 0.05).

### 3.6. Intestinal Microbiota Diversity and Microbial Composition

**Figure 4:**
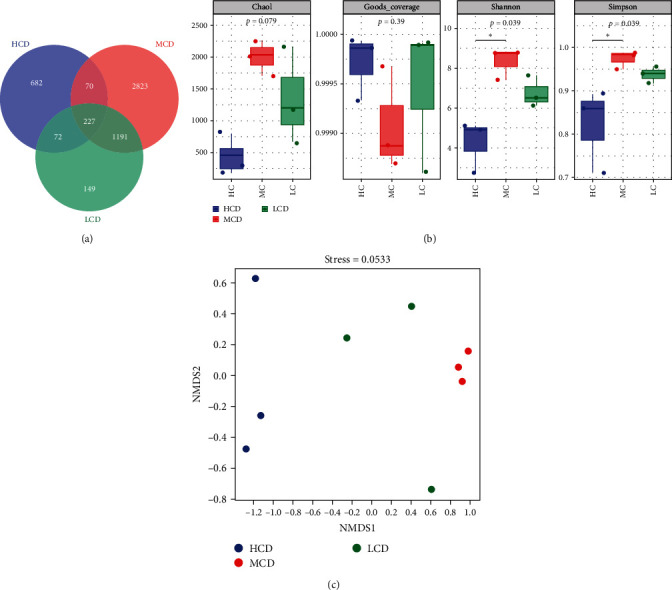
Effect of dietary carbohydrate levels on intestinal microbiota in common carp (*n* = 3). (A) Venn diagram of unique ASVs. (B) Alpha diversity: Chao1, goods coverage, Shannon, and Simpson. (C) Beta diversity: NMDS. HCD, high-carbohydrate diet; LCD, low-carbohydrate diet; MCD, medium-carbohydrate diet; NMDS, nonmetric multidimensional scaling.

The study yielded a total of 1,114,464 valid sequences at a similarity level of 97%, averaging 420 bp in length. Across the three experimental groups, there were 227 mutual ASVs (Figure 4A). The Venn diagram showed that the unique ASVs in HCD group were the lowest among the three treatment groups. Moreover, bacterial parameters related to alpha diversity in the HCD group, such as the Shannon and Simpson indices, were markedly lower compared to the MCD group (*p* < 0.05) (Figure 4B). Nonmetric multidimensional scaling (NMDS) is a ranking method based on distance or dissimilarity matrix, which aims to simplify samples or variables in high-dimensional space to low-dimensional space (usually two-dimensional or three-dimensional) for visual representation while maintaining the original distance relationship between samples as much as possible. The beta diversity index using NMDS was significantly different among the experimental groups in terms of their intestinal flora (Figure 4C).

**Figure 5:**
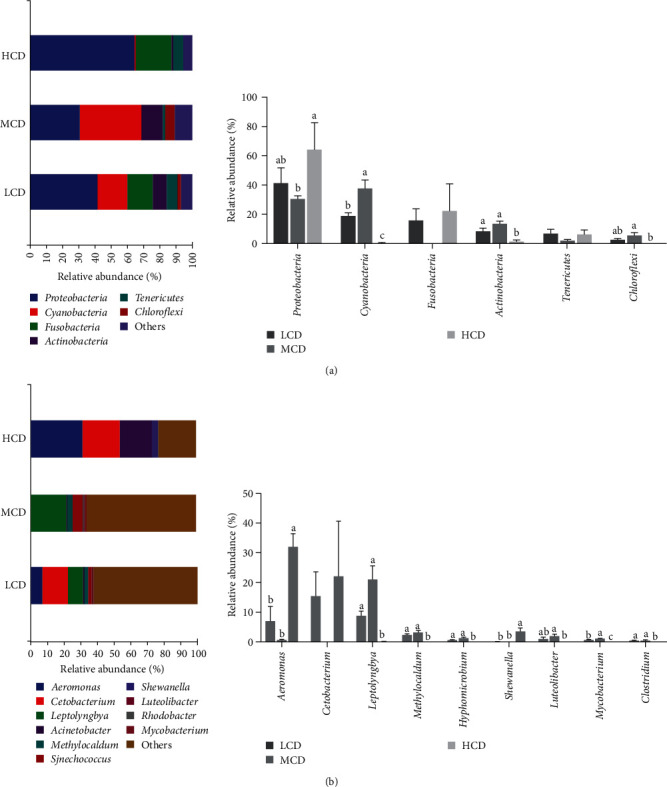
Effect of dietary carbohydrate levels on species composition of intestinal microbiota in common carp (*n* = 3): (A) at the phylum level; (B) at the genus level. HCD, high-carbohydrate diet; LCD, low-carbohydrate diet; MCD, medium-carbohydrate diet.

At the phylum level of the bacteria, *Proteobacteria*, *Cyanobacteria*, *Fusobacteria*, *Actinobacteria*, *Tenericutes*, and *Chloroflexi* were detected in all three experiment groups (Figure 5A). Among them, *Cyanobacteria*, *Actinobacteria*, and *Chloroflexi* were significantly decreased, while *Proteobacteria* were increased in HCD group (*p* < 0.05). At the genus level, the predominant bacterial flora of the HCD group includes *Aeromonas*, *Cetobacterium*, *Acinetobacter*, and *Shewanella*. In contrast, the MCD group exhibits dominant bacterial flora composed of *Leptolyngbya*, *Methylocaldum*, *Luteolibacter*, and *Mycobacterium* (Figure 5B). The genera *Aeromonas* and *Shewanella* exhibited significant upregulation in the HCD group (*p* < 0.05). On the contrary, *Leptolyngbya*, *Methylocaldum*, *Luteolibacter*, *Hyphomicrobium*, *Leptolyngbya*, and *Clostridium* were downregulated in HCD group (*p* < 0.05).

### 3.7. Intestinal Differential Metabolites

**Figure 6:**
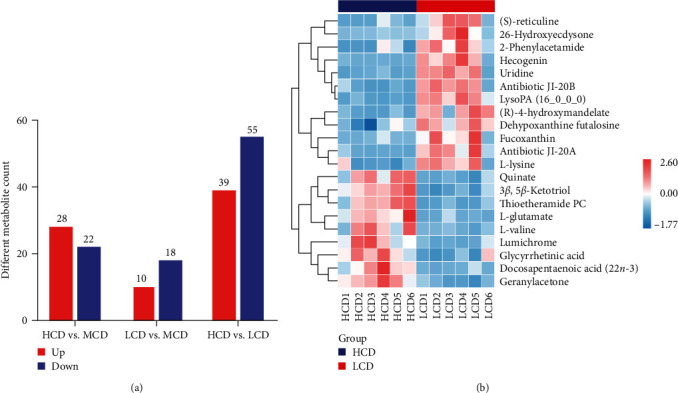
Influences of dietary carbohydrate levels on intestinal metabolites in common carp (*n* = 6): (A) the number of differential metabolites; (B) metabolic sets of HCD vs. LCD. HCD, high-carbohydrate diet; LCD, low-carbohydrate diet; MCD, medium-carbohydrate diet.

In the HCD group, 39 differential metabolites showed upregulated while 55 differential metabolites showed downregulated when compared to the LCD group (Figure 6A). Utilizing the OPLS-DA model, we performed hierarchical clustering to identify the differential metabolites with VIP > 1 and further analyzed the top 20 differential metabolites (Figure 6B). Among them, (S)-reticuline, fucoxanthin, 26-hydroxyecdysone, three antibiotic metabolites (antibiotic JI-20A, antibiotic JI-20B, 2-phenylacetamide), two nucleoside derivatives (uridine and dehypoxanthine futalosine), and three different fatty acids and derivatives (hecogenin, (R)-4-hydroxymandelate, and LysoPA (16_0_0_0)) were downregulated in HCD group. On the contrary, two amino acids (L-glutamate and L-valine), one vitamin derivative (lumichrome), one hormone compounds (3*β*, 5*β*-ketotriol), three different fatty acids and derivatives (quinate, glycyrrhetinic acid, and Docosapentaenoic acid), thioetheramide PC and and granylacetone were upregulated in HCD group.

### 3.8. Correlation Among Intestinal Microflora, Inflammation-Related Genes, and Metabolites

**Figure 7:**
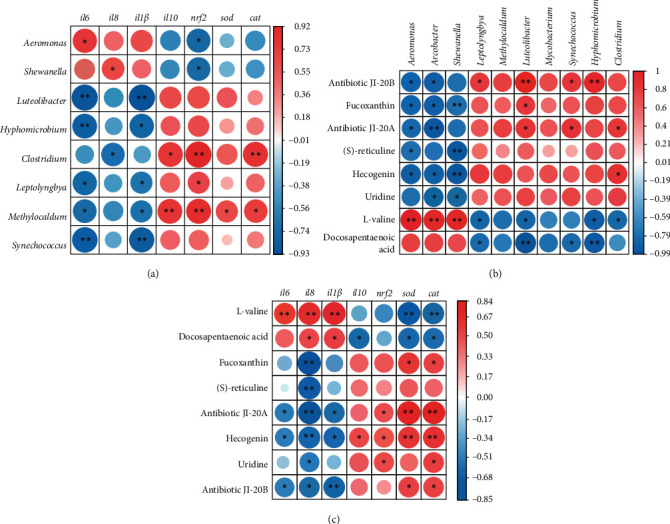
Spearman's correlation analysis. (A) The heat map showed the correlation between the gene phenotype and the enriched bacteria, comparing the HCD and LCD groups. (B) The heat map showed the correlation between the gene phenotype and the differential metabolites, comparing the HCD and LCD groups. (C) The heat map showed the correlation between the differential metabolites and the enriched bacteria, comparing the HCD and LCD groups. Each grid indicated the correlation between these attributes: red represented a positive correlation, while blue represented a negative correlation (*⁣*^*∗*^*p*  < 0.05 and *⁣*^*∗∗*^*p*  < 0.01). HCD, high-carbohydrate diet; LCD, low-carbohydrate diet.

We performed a correlation analysis to establish associations among specific metabolites, inflammation-related genes, and intestinal bacteria at the genus level. The correlation analysis results showed that intestinal microflora including *Luteolibacter*, *Hyphomicrobium*, *Leptolyngbya*, *Methylocaldum*, and *Synechococcus* had a negative correlation with pro-inflammatory factors (*il6* and *il8*) (*p* < 0.05). In addition, *Clostridium* and *Methylocaldum* had a positive correlation with *il10*, *nrf2*, and *cat* (*p* < 0.05) (Figure 7A). On the contrary, *Aeromonas* and *Shewanella* were negatively correlated with *nrf2* (*p* < 0.05). Furthermore, the metabolites such as antibiotic JI-20A, antibiotic JI-20B, and hecogenin exhibited a significant inverse correlation with *il6* and *il8*, while demonstrating a positive association with *sod* and *cat* (*p* < 0.05) (Figure 7B). On the contrary, L-valine and docosapentaenoic acid were found to have a positive correlation with pro-inflammatory factors (*il6*, *il8*, and *il1β*), while they were negatively correlated with *sod* and *cat* (*p* < 0.05). The correlation analysis of the intestinal flora with the metabolites results indicated that beneficial bacterial genera (*Luteolibacter*, *Leptolyngbya*, *Synechococcus*, and *Hyphomicrobium*) exhibited a positive correlation with antibiotic JI-20B, while showing a negative correlation with L-valine and docosapentaenoic acid (*p* < 0.05). However, pathogen genera (*Aeromonas* and *Arcobacter*) were negatively correlated with antibiotic JI-20A, antibiotic JI-20B, fucoxanthin, and hecogenin, while they were positively correlated with L-valine (*p* < 0.05) (Figure 7C).

## 4. Discussion

The present study demonstrated the influence of dietary carbohydrate level on the growth performance of common carp, with the highest growth performance in MCD group. The carbohydrate utilization capacity in omnivorous fish is better than carnivorous fish, in which 25%–40% carbohydrate is suitable for juvenile common carp [8]. This study indicated that 28% carbohydrate is in the range of carp adaptation and could be effectively used by common carp. The HSI is an essential indicator to assess hepatic degenerative changes. In this study, the HSI and VSI values were significantly increased in HCD group, which aligns with similar findings reported in juvenile giant croaker (*Nibea japonica*) [23].

Serum biochemical parameters are extensively employed for evaluating the physiological, metabolic, and nutritional status of organisms [24]. Our results indicated that HCD significantly influences glycemic content, and TG and TC levels in common carp (Table 4). It may be possibly due to the conversion of excessive dietary carbohydrates into lipids as reported previously in juvenile Furong crucian carp (*Carassius auratus* ♀×*Cyprinus carpio* ♂) [25].

The integrity of intestinal structure can guarantee the normal function of absorption and digestion. In this study, villus width in HCD group was decreased compared with MCD group. Previous research has indicated that prolonged consumption of HCDs may have a detrimental effect on intestinal structure integrity [26]. Our study demonstrated that the expression of tight junction proteins *occludin* was reduced in the HCD group (Figure 2B), which is essential for preserving intestinal barrier function [27]. This indicates that long-term intake of high-starch diet could weaken the integrity of the intestinal mucosal barrier.

Endogenous antioxidant enzymes, including SOD and CAT, play a critical role in neutralizing harmful reactive oxygen species, and the biomarker MDA reflects the level of oxidative damage in tissues and cells [28]. Our study indicated that higher carbohydrate level has resulted in decreased CAT activity and increased MDA content, indicating that a HCD leads to intestinal oxidative damage in the intestine. Furthermore, a decrease of intestinal genes such as *cat*, *sod*, and *nrf2* at the transcript levels was observed in HCD group, similar to findings in juvenile golden pompano (*Trachinotus ovatus*) [29] and Nile tilapia (*Oreochromis niloticus*) [30]. These results suggest that elevated levels of dietary carbohydrates may induce substantial oxidative damage within the organism.

The intestinal mucosa serves as a physical barrier and plays a critical role in immune defense. Maintaining immune homeostasis relies on the balance between pro-inflammatory and anti-inflammatory cytokines [26]. In this study, an increase in carbohydrate levels was found to upregulate the expression of pro-inflammatory cytokines including *il8*, *il6*, and *il1β* mRNA while downregulating the mRNA expression of *il10*. Similarly, a study in tilapia revealed that a HCD increased the mRNA expression of *il6* and *il1β* genes while decreasing the expression of *tgfβ* gene [31]. It is suggested that excessive carbohydrate intake may induce an inflammatory response.

Our study found that HCDs exerted an impact on antioxidant, immune, and growth indexes of common carp. The pivotal question arises as to whether this effect is mediated through the intricate interplay between gut microbiota and gut metabolites. It is widely recognized that greater *α*-diversity in the gut flora is linked to more extensive physiological functions, helping to maintain gut flora balance. In this study, Shannon and Simpson indices of the high-carbohydrate group were significantly decreased, indicating that elevated carbohydrate levels diminish the *α*-diversity within the intestinal flora. Similarly noteworthy findings were reported in largemouth bass (*Micropterus salmoides*) [32]. Conversely, there were no alterations in the diversity of intestinal flora within gilthead sea bream (*Sparus aurata*) when they were fed a high-starch diet [33]. This discrepancy may be attributed to various factors such as different fish species utilized or variations in feed ingredients or feeding methods.

The dominant intestinal flora at the phylum level in each experimental group were *Proteobacteria*, *Cyanobacteria*, *Fusobacteria*, *Actinobacteria*, and *Tenericutes* in our results, which are the main microbiota in common carp [34]. The core microbiota is essential for the intestinal physiological functions, including nutrient digestion, absorption, and immunity [35]. In fish, the phylum *Proteobacteria* contains many harmful bacteria species while many bacteria in phylum *Fusobacteria* are beneficial bacteria [36]. Recent study presented that the upregulation of phylum *Proteobacteria* may lead to intestinal inflammation and obesity [37]. Our study found a significant correlation between the expression of pro-inflammatory factor genes and the abundance of *Proteobacteria*, indicating a potential link between the presence of *Proteobacteria* and the inflammatory response. Specifically at the genus level, the LCD group showed significantly lower abundances of opportunistic pathogenic bacteria, including *Aeromonas*, *Sheverella*, and *Acinetobacter* in the phylum *Proteobacteria*. This indicates that a HCD may be likely to nourish harmful gut bacteria. These results align with similar observations in largemouth bass, confirming that dietary carbohydrate levels can impact the gut microbiota composition [38]. Therefore, high carbohydrate levels promote the growth of harmful bacteria, while low carbohydrate levels favor the growth of beneficial bacteria, which may potentially influence overall health in fish.

The intestinal microbiota exerts an influence on host development, health, and pathogenesis by metabolizing ingested nutrients into specific metabolites. These metabolites then act as information messengers between the intestinal microbiota and host cells [39]. The LCD group showed a significant increase in anti-inflammatory active substances (fucoxanthin, (S)-reticuline, hecogenin, antibiotic JI-20A, and antibiotic JI-20B) (Figure 6B) compared to the HCD group. Moreover, arachidonic acid metabolism has previously been confirmed to be closely associated with inflammation. In this study, the HC group exhibited markedly higher levels of docosapentaenoic acid (22*n*-3) in comparison to the LCD group. It has been documented that elevated dose of PUFA have the potential to exacerbate inflammation [40]. In summary, dietary carbohydrates could potentially modulate gut microbiota and their metabolites, thereby exerting an influence on intestinal inflammatory response.

Furthermore, the present study reveals a noteworthy finding regarding the association between differential metabolites and intestinal flora. Notably, a negative correlation is observed between the anti-inflammatory-active substances (fucoxanthin, reticuline, 2-phenylacetamide, hecogenin, antibiotic JI-20A, and antibiotic JI-20B) and the bacteria *Aeromonas*, *Rubellimicrobium*, and *Arcobacter*. Conversely, these substances display a positive correlation with other bacterial types such as *Luteolibacter* and *Methylosinus*. These results emphasize the intricate types of bacterial interactions in the presence of these anti-inflammatory compounds. In a study conducted on Indian major carp, rohu *Labeo rohita*, it was observed that an increased abundance of *Luteolibacter* can effectively restore the intestinal barrier [41].

## 5. Conclusion

In summary, the strategic addition of carbohydrates to the carp diet has been shown to enhance their growth and increase the prevalence of beneficial intestinal bacteria. On the other hand, excessive carbohydrate intake hampers the growth of common carp and worsens both harmful bacterial colonization and pro-inflammatory factor expression. Notably, certain anti-inflammatory factors, such as fucoxanthin, (S)-reticuline, 2-phenylacetamide, hecogenin, antibiotic JI-20A, and antibiotic JI-20B, have significant correlations with *Luteolibacter*, highlighting the complex interactions and dependencies within this biological system. In summary, dietary carbohydrates may regulate the intestinal flora, and their metabolites, and influence the intestinal inflammatory response.

## Figures and Tables

**Table 1:** Ingredient composition and nutrient content of experimental diets.

Ingredients (%)	Groups		
	LCD	MCD	HCD
Casein	20	20	20
Fish meal	20	20	20
Gelatin	5	5	5
Pregelatinized starch	15	28	45
Soybean oil	5	5	5
Ca(H_2_PO_4_)_2_	2.5	2.5	2.5
Choline chloride	0.2	0.2	0.2
Zeolite powder	0.81	0.81	0.81
CMC^a^	1	1	1
Vitamin premix^b^	0.08	0.08	0.08
Mineral premix^c^	0.11	0.11	0.11
Microcrystalline cellulose	30.3	17.3	0.3
Total	100	100	100
Proximate composition (percentage in dry matter)			
Dry matter	94.85	94.75	94.85
Crude protein	35.28	35.31	35.37
Crude lipid	6.61	6.63	6.67
Ash	6.76	6.68	6.76
Crude fiber	31.21	18.34	1.51
Digestibility carbohydrates	14.99	27.79	44.54
Gross energy^d^ (MJ/kg)	13.52	15.73	18.64

Abbreviations: HCD, high-carbohydrate diet; LCD, low-carbohydrate diet; MCD, medium-carbohydrate diet.

^a^CMC, carboxymethylcellulose.

^b^Vitamin premix (mg/kg premix): Vitamin A, 6,000,000 IU; Vitamin D_3_, 2,000,000 IU; Vitamin E, 30,000 mg; Vitamin K_3_, 9000 mg; Vitamin B_1_, 1800 mg; Vitamin B_2_, 2880 mg; Vitamin B_6_, 18,000 mg; Vitamin B_12_, 20 mg; Vitamin C, 20,000 mg; niacinamide, 42,000 mg; folic acid, 3000 mg; calcium pantothenate, 18,000 mg; biotin, 60 mg; and phaseomannite, 24,000 mg.

^c^Mineral premix (mg/kg premix): Cu, 12,000 mg; Fe, 20,000 mg; Zn, 32,000 mg; Mn, 40,000 mg; I, 800 mg; and Se, 400 mg.

^d^Gross energy = 23.6 kJ/g protein + 39.5 kJ/g lipid + 17.2 kJ/g and 17.2 kJ/g digestible carbohydrates.

**Table 2:** Real-time PCR primer sequences.

Gene	Sequence (5′–3′)	GenBank ID
*sod* F	CGCACTTCAACCCTCAT	XM_019111527.2
*sod* R	CATTGCCTCCTTTACCC	
*cat* F	TTCCTGTGGGACGCCTTGT	JF411604.1
*cat* R	TCCGAGCCGATGCCTATGT	
*keap1* F	CAGTGGGCGAGAAGTGT	JX470752.1
*keap1* R	TTTGATGGCTCCAGGTT	
*nrf2* F	ACGACAAATGCCGAAGT	JX462955.1
*nrf2* R	CTGCCTCATCTAGTGGAAA	
*il1β* F	TTACAGTAAGACCAGCCTGA	XM_019080073.2
*il1β* R	AGGCTCGTCACTTAGTTTGT	
*il6* F	GCAGCGCATCTTGAGTGTTTAC	XM_019110666.2
*il6* R	CTGCTGCTCCATCACTGTCTTC	
*il8* F	CCTGACCACTGGTGAAGGAA	KU881637.1
*il8* R	GGTGGCA ATGATCTCTGTGTCT	
*il10* F	CTCCGTTCTGCATACAGAGAAA	XM_019092454.1
*il10* R	TCATGACGTGACAGCCATAAG	
*tgf-β* F	ACGCTTTATTCCCAACCAAA	AF136947.1
*tgf-β* R	GAAATCCTTGCTCTGCCTCA	
*nf-κb* F	AATGTGGTGCGTCTGTGCTT	XM_019094112.1
*nf-κb* R	TGTTGTCATAGATGGGGTTGGA	
*occludin* F	CAGGAGGCATCCATGGTGTT	XM_042729457.1
*occludin* R	AAGACACTGCCGGTCTTCAG	
*zo-1* F	CCGAAGCTTTGACAGCAAAC	XM_0427600882.1
*zo-1* R	GGTTGATCTTCCACTGACTC	
*claudin-1* F	CTGGAGTTGATGGGTTTCTTTCTTTTG	XM_042749400.1
*claudin-1* R	AGACCTTTCATGCTTTCTACCG	
*18s* F	GAGACTCCGGCTTGCTAAAT	FJ710826.1
*18s* R	CAGACCTGTTATTGCTCCATCT	

Abbreviations: *18 s*, 18 s ribosomal RNA*; cat*, catalase; *il1β*, interleukin 1 beta; *il6*, interleukin 6; *il8*, interleukin 8; *il10*, interleukin 10; *keap1*, kelch-like ECH-associated protein 1; *nf-κb*, nuclear factor-kappa b; *nrf2*, nuclear factor erythroid-2-related factor 2; *sod, superoxide dismutase; tgf-β, transforming growth factor-beta; zo-1*, zonula occludens protein 1.

**Table 3:** Growth performance of common carp with different carbohydrate levels.

Parameters	Groups			F statistic	*p*-Value
	LCD	MCD	HCD		
IBW (g)	8.81 ± 0.01	8.82 ± 0.01	8.82 ± 0.01	0.267	0.775
FBW (g)	84.42 ± 1.90^b^	90.63 ± 1.77^a^	88.31 ± 2.00^ab^	2.746	0.066
FCR	1.06 ± 0.28^a^	0.97 ± 0.23^b^	1.01 ± 0.15^ab^	3.266	0.040
WGR (%)	858.34 ± 21.55^b^	927.53 ± 20.03^a^	901.23 ± 22.73^ab^	2.655	0.072
SGR (%/day)	3.50 ± 0.04^b^	3.61 ± 0.03^a^	3.56 ± 0.04^ab^	0.583	<0.001
CF (g/cm^3^)	2.34 ± 0.03^b^	2.38 ± 0.03^ab^	2.45 ± 0.02^a^	4.083	0.018
VSI (%)	7.68 ± 0.52^b^	9.30 ± 0.41^ab^	9.32 ± 0.45^a^	4.073	0.026
HSI (%)	1.96 ± 0.12^c^	2.63 ± 0.17^b^	3.38 8± 0.19^a^	19.569	<0.001
SR (%)	100.00 ± 0.00	98.89 ± 1.11	98.89 ± 1.11	0.500	0.630

*Note:* The values were reported as means ± SEM (*n* = 3). Different superscripts in the same row indicate a statistically significant difference (*p* < 0.05). FCR = Feed consumed/weight gain; WGR = 100 × (FBW − IBW)/IBW; SGR (%/day) = 100 × (Ln final individual weight − ln initial individual weight)/number of feeding days; CF (g/cm^3^) = 100 × body weight/body length^3^; VSI (%) = 100 × viscera wet weight/body wet weight; HSI (%) = 100 × hepatosomatic wet weight/body wet weight; and SR (%) = 100 × (final number of fish)/(initial number of fish).

Abbreviations: CF, condition factor; FBW, final body weight (g); FCR, feed conversion ratio; HCD, high-carbohydrate diet; HSI, Hepatosomatic index; IBW, initial body weight (g); LCD, low-carbohydrate diet; MCD, medium-carbohydrate diet; SGR, specific growth rate; SR, survival rate; VSI, viscero somatic index; WGR, weight gain rate.

**Table 4:** Serum biochemical indicators of common carp with different carbohydrate levels.

Parameters (mmol/L)	Groups		
	LCD	MCD	HCD
GLU	6.62 ± 0.54^b^	6.86 ± 0.73^b^	9.47 ± 0.79^a^
Lactate	8.82 ± 0.50	9.96 ± 0.55	9.80 ± 1.00
TG	1.15 ± 0.07^b^	1.74 ± 0.13^a^	1.63 ± 0.13^a^
TC	3.76 ± 0.27^b^	4.59 ± 0.22^a^	4.23 ± 0.23^ab^
HDL-C	1.35 ± 0.20^b^	2.39 ± 0.17^a^	2.02 ± 0.11^a^
LDL-C	2.37 ± 0.19	2.66 ± 0.31	2.60 ± 0.20

*Note:* Means ± SEM per diet in triplicate tanks (*n* = 12) were compared using a one-way ANOVA analysis followed by Duncan's test (*p*  < 0.05), with different letters indicating significant differences between the diets.

Abbreviations: GLU, glucose; HCD, high-carbohydrate diet; HDL-C, high-density lipoprotein-cholesterol; LCD, low-carbohydrate diet; LDL-C, low-density lipoprotein-cholesterol; MCD, medium-carbohydrate diet; TC, total cholesterol; TG, triglyceride.

**Table 5:** Effects of dietary carbohydrate levels on intestine morphology parameters of common carp.

Items (μm)	Groups		
	LCD	MCD	HCD
Villus height	289.78 ± 4.99^b^	355.34 ± 5.40^a^	347.57 ± 4.00^a^
Villus width	59.04 ± 1.47^b^	72.28 ± 1.78^a^	60.51 ± 1.34^b^
Muscle layer thickness	39.72 ± 1.76^b^	45.98 ± 1.40^a^	45.46 ± 0.91^a^

*Note:* Different letters in the same row indicate significant differences (*p*  < 0.05).

Abbreviations: HCD, high-carbohydrate diet; LCD, low-carbohydrate diet; MCD, medium-carbohydrate diet.

## Data Availability

Data will be made available upon request.

## Supporting Information

Supporting Information Graphical abstract: Long-term feeding of high-carbohydrate diets to common carp has been shown to increase the abundance of harmful bacteria such as *Aeromonas*, *Shewanella*, and *Rheinheimera* in the intestine. This shift in microbial composition leads to the production of pro-inflammatory substances, including quinate and L-valine, and subsequently reduces the intestinal antioxidant capacity. Conversely, feeding these carp a low-carbohydrate diet enhances the abundance of beneficial bacteria, including *Leptolyngbya*, *Mycobacterium*, *Methylocaldum*, and *Clostridium*. This beneficial microbial shift results in the production of anti-inflammatory substances such as hecogenin and uridine, thereby improving the intestinal antioxidant capacity.
