# Supplementary material for: The Effect of High Levels Carbohydrate on Intestinal Microbiota, Metabolites, and Health of Common Carp (Cyprinus carpio L.)
Source: Aquac Nutr. 2024 Oct 23;2024:7631021. doi: 10.1155/2024/7631021 (PMC11524719; doi:10.1155/2024/7631021)

**Graphical abstract：**Long-term feeding of high-carbohydrate diets to common carp has been shown to increase the abundance of harmful bacteria such as *Aeromonas*, *Shewanella*, and *Rheinheimera* in the intestine. This shift in microbial composition leads to the production of pro-inflammatory substances, including Quinate and L-Valine, subsequently reducing the intestinal antioxidant capacity. Conversely, feeding these carp a low-carbohydrate diet enhances the abundance of beneficial bacteria such as *Leptolyngbya*, *Mycobacterium*, *Methylocaldum*, and *Clostridium*. This beneficial microbial shift results in the production of anti-inflammatory substances like Fucoxanthin, (S)-Reticuline, Hecogenin and Uridine, thereby improving the intestinal antioxidant capacity.


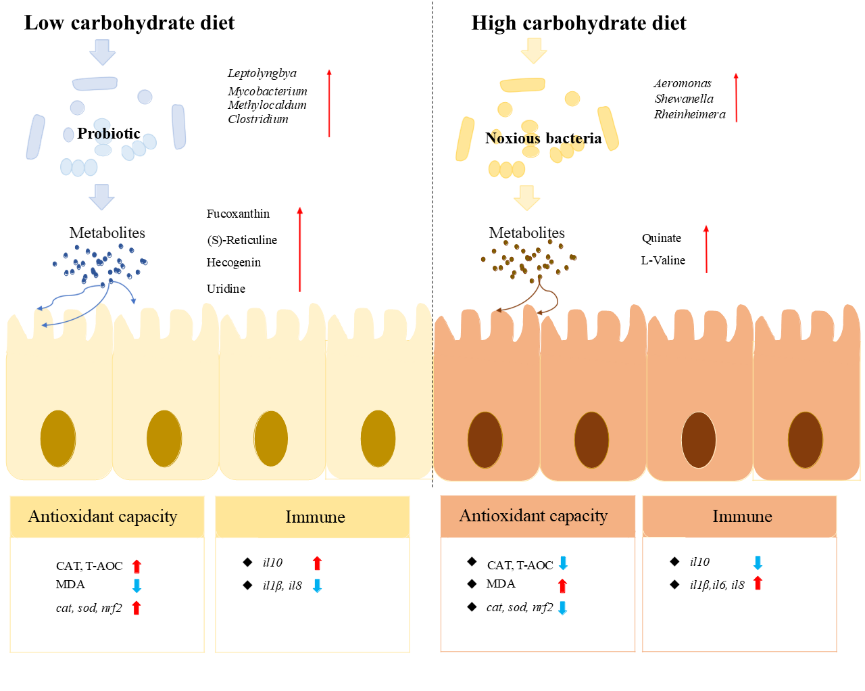

Supplement: Supporting Information — Graphical abstract: Long-term feeding of high-carbohydrate diets to common carp has been shown to increase the abundance of harmful bacteria such as Aeromonas, Shewanella, and Rheinheimera in the intestine. This shift in microbial composition leads to the production of pro-inflammatory substances, including quinate and L-valine, and subsequently reduces the intestinal antioxidant capacity. Conversely, feeding these carp a low-carbohydrate diet enhances the abundance of beneficial bacteria, including Leptolyngbya, Mycobacterium, Methylocaldum, and Clostridium. This beneficial microbial shift results in the production of anti-inflammatory substances such as hecogenin and uridine, thereby improving the intestinal antioxidant capacity. [file 7631021.f1.docx]
